# Traditional Chinese medicine tonifying kidney therapy (Bu shen) for stable chronic obstructive pulmonary disease: Protocol for a systematic review and meta-analysis

**DOI:** 10.1097/MD.0000000000013701

**Published:** 2018-12-28

**Authors:** Gao Zhen, Liu Yingying, Dong Jingcheng

**Affiliations:** Institutes of Integrative Medicine, Fudan University, Shanghai, China.

**Keywords:** chronic obstructive pulmonary disease, clinical efficacy, protocol, systematic review, tonifying kidney therapy (Bushen), traditional Chinese medicine

## Abstract

**Background:**

Traditional Chinese medicine (TCM) is commonly used to combine with pharmacotherapy for stable chronic obstructive pulmonary disease (COPD) in China and other Asian countries such as South Korea and Japan. The objective of this systematic review is to evaluate the efficacy and safety of tonifying kidney therapy (Bushen, TK) for stable COPD.

**Methods:**

Randomized controlled trials (RCTs) of TK for stable COPD were searched from 4 databases including Pubmed, the Cochrane library, CBM (China Biology Medicine disc, CBMdisc), CNKI (China National Knowledge Infrastructure) from inception to December 2017. Two reviewers independently screened the literature, extracted the data and assessed the risk of bias in included studies. RevMan 5.3 software was used for meta-analysis. The primary outcomes analyzed in this meta-analysis were effectiveness, TCM Syndrome Score, dyspnea (modified Medical Research Council questionnaire [mMRC]), COPD health status (COPD Assessment Test [CAT]), exercise capacity (6-min walk distance in meters [6mWD]), and respiratory-specific quality of life (St George's Respiratory Questionnaire [SGRQ]). Second outcomes analyzed for this meta-analysis were lung function (forced expiratory volume in 1 second [FEV1], FEV1%, forced vital capacity [FVC], FEV1/FVC), the frequency of acute exacerbation, T-lymphocyte subsets (CD4, CD8, CD4/CD8), and immunoglobulin (IgA, IgG, and IgM). The summary results will be pooled using the random-effects model or fixed-effects model according to the heterogeneity of the included studies.

**Result:**

This systematic review will provide an evidence of TK for stable COPD, and will submit to a peer-reviewed journal for publication.

**Conclusion:**

The conclusion of this systematic review will provide evidence to judge whether TK is an effective intervention for stable COPD patients.

**PROSPERO registration number:**

PROSPERO CRD 42018090328.

## 1 Introduction

Chronic obstructive pulmonary disease (COPD) is a disorder characterized by progressive airflow limitation caused by chronic inflammation in airways and lung parenchyma. COPD is generally associated with symptoms such as cough, sputum production, and dyspnea.^[1]^ COPD has been a major public health problem in the 21^st^ century,^[2]^ that imposes a substantial economic burden on both patients and government in China.^[3]^ Patients who suffer from COPD may experience cough, dyspnea, chest tightness, and wheezing.^[4,5]^ Chronic cough, sputum production and decreased forced expiratory volume in 1 second (FEV1) have proved to be independently associated with an increased risk of frequent exacerbations and hospitalizations.^[6]^ Pharmacologic therapies and nonpharmacologic therapies are frequently used to manage COPD as recommended by the World Health Authority (WHO) and GOLD.^[7]^ Nevertheless, it is still unclear whether these therapies can suppress the progression of this disease.

Traditional Chinese medicine (TCM) has a long history and is a common aspect of the healthcare system in many Asian countries. COPD belongs to the category of lung distention (Feizhang disease) in TCM.^[8]^ Considering that COPD is a chronic disease, researchers should develop medical therapy that can be used continuously for symptom control. Also, treatment of patients in stable phase is very important for the outcome of COPD. TCM therapies have shown the ability to improve symptoms, reduce the frequency of acute exacerbation and to improve the quality of life in stable COPD.^[9]^ In TCM theory, “the essence of Kidney (Shen) and Qi” is considered a core concept.^[10]^ The syndrome of lung (Fei)-kidney *(*Shen) qi deficiency is one of the most common syndromes in stable COPD;^[7]^ kidney (Shen) qi deficiency is the primary TCM pathogenesis in stable COPD.^[11]^ Tonifying kidney therapy (Bushen, TK) is a TCM mixture of several compounds designed to tonifying kidney (Shen), which is widely used in combination with other TCM therapies and/or conventional western medicine (CWM) in China for the clinical treatment of stable COPD.^[12,13]^ TK treatments for COPD, include tonifying kidney (Shen) only, tonifying kidney (Shen) and lung (Fei), tonifying kidney (Shen) and spleen (Pi), and tonifying lung (Fei), spleen (Pi) and kidney (Shen).

Nevertheless, data supporting the validity of this treatment are insufficient. This systematic review aimed to evaluate the efficacy and safety of TK as a treatment for stable COPD by integrating different outcomes from RCTs.

## 2 Materials and methods

### 2.1 Registration

The study protocol has been registered on international prospective register of systematic review (PROSPERO). The study registration number of PROSPERO is CRD 42018090328. The procedure of this protocol will be conducted according to the preferred reporting items for systematic review and meta-analysis protocols (PROSMA-P) guidance.^[14]^

### 2.2 Inclusion and exclusion criteria

All the RCTs reporting the application of TK for the treatment of stable COPD were included. There were no limitations on publication status. The inclusion criteria were the following:

1) article published in English or Chinese language;
2) randomized or quasi-randomized clinical trials;
3) studies including patients diagnosed with stable COPD;
4) studies including patients treated according to syndrome differentiation (TCM).

The exclusion criteria were:

1) randomized crossover trials, case reports, case series, reviews, qualitative studies, or animal experiments;
2) stable COPD interventions being combined with external therapy of TCM.

### 2.3 Interventions type

RCTs that examined the effects of TK combined with the CWM and CWM were identified. Patients in treatment group were given TK combined with CWM, while patients in control group were treated only with CWM. Patients were excluded when the RCTs included external therapy of TCM. We did not set limitations on dosages and course of treatment.

### 2.4 Outcome measures

The primary outcomes analyzed in this meta-analysis were effectiveness, TCM Syndrome Score,^[15]^ dyspnea (modified Medical Research Council questionnaire [mMRC]),^[16]^ COPD health status (COPD Assessment Test [CAT]),^[17]^ exercise capacity (6-min walk distance in meters [6mWD]),^[18]^ and respiratory-specific quality of life (St George's Respiratory Questionnaire [SGRQ]).^[19]^ Second outcomes analyzed for this meta-analysis were lung function (FEV1, FEV1%, forced vital capacity [FVC], FEV1.FVC), the frequency of acute exacerbation, T-lymphocyte subsets (CD4, CD8, CD4/CD8) and immunoglobulin (IgA, IgG, and IgM).

### 2.5 Literature search strategy

Two Chinese language databases and 2 English language databases were widely searched for all relevant results until December 2017 by Gao Zhen and Liu Yingying. The Chinese language databases were China National Knowledge Infrastructure (CNKI) and China Biology Medicine disc (CBM). The 2 English language databases were PubMed and Cochrane Library.

Search strategy was the following: #1 Bushen; #2 Bu shen; #3 Yishen; #4 nourishing the kidney; #5 tonifying the kidney; #6 Yi shen; #7 tonifying shen; #8 tonifying kidney; #9 nourishing kidney; #10 nourishing shen; #11 reinforcing the kidney; #12 reinforcing kidney; #13 reinforcing shen; #14 Invigorating the kidney; #15 Invigorating kidney; #16 Invigorating shen; #17 kidney-reinforcin; #18 kidney reinforcing; #19 Shen reinforcing; #20 Shen-reinforcing; #21 kidney-Invigorating; #22 kidney Invigorating; #23 Shen-Invigorating; #24 kidney-tonifying; #25 Shen-tonifying; #26 kidney tonifying; #27 Shen tonifying; #28 Shen Invigorating; #29 Invigorating Shen; #30 #1 or #2 or #3 or #4 or #5 or #6 or #7 or #8 or #9 or #10 or #11 or #12 reinforcing kidney or #13 or #14 or #15 or #16 or #17 or #18 or #19 or #20 or #21or #22 or #23 or #24 or #25 or #26 or #27 or #28 or #29; #31 COPD; #32 COPD; #33 #31 or #32; #34 #30 and #33

### 2.6 Selection of studies

Two researchers (Gao Zhen, Liu Yingying) will scan the titles and summary of the articles they get based on an inclusion criterion that is made previously to eliminate some uncorrelated documents. Besides, for the documents that fit the inclusion criteria, the valuators will read the whole article to make sure if they meet a criterion and prepare to extract relevant information, check the result of the documents brought in. If it meets any diverges, the problem will be solved by consulting another researcher (Dong Jingcheng). The lacking information will be replenished by contacting with the writer of the original article.

### 2.7 Data extraction and management

Data were extracted by 2 reviewers independently (Gao Zhen, Liu Yingying). After checking, any disagreements were resolved by consulting a third reviewer (Dong Jingcheng). All the data were recorded using a data collection form. The form contents were as follows:

1) Title, authors, source, and time of publication;
2) Basic characteristics (Sample, gender, age, diagnostic criteria, course of disease, intervention, course of treatment, main outcomes, specific details);
3) Methods (study design, total study duration, sequence generation, allocation sequence concealment, and blinding, other concerns about bias).

The collected outcome data was inputted into Review Manager 5.3 (RevMan5.3).

### 2.8 Assessment of risk of bias

Criteria for judging the risk of bias were taken from the “risk of bias” assessment tool in The Cochrane Handbook for Systematic Reviews of Interventions 5.1.0.^[20]^ This judgment was evaluated by 2 reviewers (Gao Zhen, Liu Yingying) independently, and the disagreements were resolved by consulting a third reviewer (Dong Jingcheng).

### 2.9 Data synthesis

RevMan 5.3 was used for statistical analysis. The extracted data were divided into dichotomous and continuous variables. Data were summarized using Odds ratio (OR) with 95% confidence intervals (CI) for dichotomous outcomes; mean difference (MD) with 95% CI was presented for continuous outcomes. Cochrane's *P* values and *I*^2^ tests were determined to examine the level of heterogeneity between trials. A random-effects model was used to evaluate the effects of TK on stable COPD if *I*^2^ > 50% or *P* <.1. Otherwise, a fixed-effects model was utilized. *P* <.01 was considered statistically significant. Data were subjected to meta-analysis by using Review Manager 5.3 (Cochrane Community, London, United Kingdom, 2014). Sensitivity analysis was performed to assess the stability of conclusions. Where heterogeneity was detected, accepted methods were used to explore the statistical heterogeneity using clinical parameters such as treatment duration, sample size, publication year, diagnostic criteria, and publication language. Publication bias was analyzed by funnel plot analysis if sufficient studies (n ≥10) were found.

TK in treating stable COPD including tonifying kidney (Shen) only, tonifying kidney (Shen) and lung (Fei), tonifying kidney (Shen), and spleen (Pi), and tonifying lung (Fei), spleen (Pi) and kidney (Shen). So subgroup analysis will be performed on the basis of those different interventions.

### 2.10 Quality of evidence

We will also assess the quality of evidence for the main outcomes with the Grading of Recommendations Assessment, Development, and Evaluation approach. The 5 items will be investigated, including limitations in study design, inconsistency, inaccuracies, indirectness, and publication bias.

### 2.11 Ethics and dissemination

This systematic review will not require ethical approval because there are no data used in our study that are linked to individual patient data. In addition, findings will be disseminated through peer-review publications.

## 3 Discussion

In 2007, the prevalence of COPD was 8.2% in people ≥40 years of age in China (Beijing, Tianjin, Liaoning, Shanghai, Guangdong, Shanxi and Chongqing).^[21]^ According to meta-analysis, the prevalence of COPD in China between 2000 to 2014 was 9.3% in people ≥40 years of age.^[22]^ COPD was detected in 13.4% of subjects aged ≥40years (19.4% of men and 7.9% of women) in Korea. ^[23]^ COPD was the fifth cause of death in 2002 and it is projected to be the fourth cause of mortality by 2030.^[24]^ Therefore, the effective prevention and treatment of COPD continue to remain a vital public health concern to be urgently resolved. Many guidelines have been developed,^[25,26]^ like a combination of smoking cessation, vaccination, pharmacologic therapy and physical activity, and proper management of COPD exacerbations (AECOPD) with pharmacologic therapy. Oral systemic corticosteroids, theophylline, and some classes of long-acting inhaled therapeutic agents such as long-acting β-agonist plus inhaled corticosteroid are commonly used in the treatment of COPD.^[27]^ However, there are still difficulties in helping people control their symptoms as well as eliminating medication-induced side effects or adverse events.^[28]^

In China and other Asian countries such as South Korea and Japan, COPD patients are seeking TCM management when western medical treatment is not sufficient to maintain their quality of daily work and life. So far, many complementary and alternative medicines have been assessed with promising results.^[29]^ The Chinese terms Fei-Zhang (Lung distension) and Chuan-Zheng (panting) have traditionally been used to describe symptoms of COPD.^[30]^ TCM has long been used for the treatment of Feizhang disease (COPD) in China.

According to TCM, being the source of growth and development the spleen (Pi) provides the material basis for the acquired constitution, while the kidney (shen) as the origin of congenital constitution stores vital essence and energy. Mutual generation between lung and kidney, that is, spleen (Pi) and kidney (Shen) deficiency, is the basis of the incidence of COPD. Lung (Fei) and kidney (Shen) deficiency are the main features of stable COPD, whereas the kidney (Shen) deficiency is the root cause. For stable COPD, TK is one of the most important treatments aiming at its pathogenesis.^[31]^ In TCM clinical practice and in some clinical trials, TK is used in treatment of stable COPD without TCM syndrome differentiation-typing and have been shown to have certain effect.^[32,33]^ TK in treating stable COPD including tonifying kidney (Shen) only, tonifying kidney (Shen) and lung (Fei), tonifying kidney (Shen) and spleen (Pi), and tonifying lung (Fei), spleen (Pi) and kidney (Shen). So subgroup analysis will be performed on the basis of those 4 different interventions.

This study focuses on evaluating the efficacy and safety of TK in combination of CWM for treating COPD compared with CWM alone. It may help to propose the clinical recommendation for stable COPD patients and to provide more reliable evidence for TK's application.

## Author contributions

**Data curation:** Gao Zhen, Liu Yingying.

**Formal analysis:** Gao Zhen, Liu Yingying.

**Methodology:** Gao Zhen.

**Project administration:** Dong Jingcheng.

**Resources:** Gao Zhen, Liu Yingying.

**Software:** Gao Zhen.

**Visualization:** Gao Zhen, Liu Yingying.

**Writing – original draft:** Gao Zhen, Liu Yingying.

**Writing – review & editing:** Gao Zhen, Dong Jingcheng.

**Data curation:** Zhen Gao, Liu Yingying.

**Formal analysis:** Zhen Gao, Liu Yingying.

**Methodology:** Zhen Gao.

**Project administration:** Dong Jingcheng.

**Resources:** Zhen Gao, Liu Yingying.

**Software:** Zhen Gao.

**Visualization:** Zhen Gao, Liu Yingying.

**Writing – original draft:** Zhen Gao, Liu Yingying.

**Writing – review & editing:** Zhen Gao, Dong Jingcheng.
